# Can Neutrophil-Lymphocyte Ratio, Platelet-Lymphocyte Ratio, or Systemic Immune Inflammation Index Be an Indicator of Postoperative Pain in Patients Undergoing Laparoscopic Cholecystectomy?

**DOI:** 10.7759/cureus.33955

**Published:** 2023-01-19

**Authors:** Şenay Canikli Adıgüzel, Dilan Akyurt, Hatice Bahadır Altun, Serkan Tulgar, Gökçe Ültan Özgen

**Affiliations:** 1 Anesthesiology and Reanimation, Samsun University Faculty of Medicine Samsun Training and Research Hospital, Samsun, TUR; 2 Anesthesiology, Samsun University Faculty of Medicine Samsun Training and Research Hospital, Samsun, TUR

**Keywords:** systemic immune inflammation score, platelet lymphocyte ratio, neutrophil lymphocyte ratio, postoperative analgesia, laparoscopic cholecystectomy

## Abstract

Aim: Through this study, we aim to investigate whether biomarkers like the neutrophil-lymphocyte ratio (NLR), platelet lymphocyte ratio (PLR), and systemic immune-inflammatory index (SII) might predict the postoperative first 24 hours analgesic requirement and pain scores of patients undergoing laparoscopic cholecystectomy.

Material & Method: After receiving the local ethical board approval, records of 67 patients, aged between 18 and 75 years, with ASA classifications I-III who underwent elective laparoscopic cholecystectomy were retrospectively evaluated to record postoperative analgesic requirements and numerical pain scale (NRS) scores. NLR, PLR, and SII scores were calculated from preoperative hemograms and compared with analgesic requirements and NRS scores.

Results: The data of 67 patients were evaluated. There was no correlation between postoperative tramadol use and NRS scores, PLR, or SII values (p>0.05). NRS scores and the cumulative 24-hour postoperative tramadol use were correlated (p=0.0001), as it was observed that patients with high NRS scores used higher amounts of tramadol. Additionally, a poor statistically significant correlation was found between PDW (Platelet distribution width) value and tramadol dose (AUC = 0.611).

Conclusion: No significant association between NLR, PLR, SII, pain scores, and tramadol use was detected.

## Introduction

The first laparoscopic cholecystectomy was performed in France in 1987, and it quickly became an alternative to open cholecystectomy throughout the world. It is a safe and efficient treatment for symptomatic gallstones [1]. Laparoscopic interventions, which are frequently utilized nowadays, provide considerable advantages over conventional surgery, including reduced surgical trauma, a shorter hospital stay, and a faster functional recovery. Of these interventions, laparoscopic cholecystectomy (LC) is one of the most frequently performed. The pain experienced after laparoscopy is different from the experienced pain after laparotomy. In contrast to laparotomy, in which parietal (abdominal wall) discomfort predominates, laparoscopic procedures are also associated with visceral pain. In laparoscopic procedures, in addition to surgical trauma, the local irritation of intraperitoneal carbon dioxide and the increase in intra-abdominal pressure contribute to postoperative pain [2].

Postoperative pain occurs as an inflammatory reaction to surgical trauma. Incisions, dissection, retraction, and other surgical interventions lead to a local inflammatory mediator response, leading to an increase in nociceptor sensitivity and hyperalgesia, resulting in a sense of postoperative pain [3]. Numerous studies have shown that postoperative pain is influenced by variables like the type of operation, presence of preoperative pain, demographic features, and physiological factors [4]. Surgical trauma induces the acute phase response, which controls tissue damage, limits infection, and initiates the healing process. During the inflammatory reaction, the blood's leukocyte count also changes [4].

Neutrophil/Lymphocyte Ratio (NLR) is a simple and inexpensive inflammatory response marker that has become increasingly popular in recent years [4-6]. Also utilized are the Platelet/Lymphocyte ratio (PLR) and the systemic immunological inflammation score (SII). These indicators, calculated from regular hemogram parameters, are affordable and readily available biomarkers that have been found to be useful in the differential diagnosis of various diseases, illness studies, and therapy response evaluation [5].

Our goal in this study is to determine whether there is a correlation between hemogram parameters and parameters obtained from hemograms, pain scores, and analgesic requirements in the postoperative 24-hour period in patients who have undergone LC surgery, and whether these have predictive value for pain conditions.

## Materials and methods

This retrospective study was registered on ClinicalTrials.gov (NCT05558553) following approval by the local ethics committee (SUKAEK) on 29 June 2022 with decision number 2022/4/11.

The records of patients aged 18 to 75 who underwent elective LC surgery between January 1, 2022, and August 31, 2022, and had ASA scores of I-III were evaluated using the hospital automation system (Fonet v4.22.10.1) and patient files. The study excluded individuals with hematological disease, immunosuppressive therapy, inflammatory disease, gastrointestinal tumor, rheumatic disease, uncontrolled diabetes, and chronic organ failure, as well as those who were corticosteroid and opioid users, obese, and who had open surgery. Those who had recently experienced cholecystitis or pancreatitis were also excluded. Age, gender, comorbidities, duration of surgery, presence of surgical complications, and hemodynamic and respiratory problems were extracted from the patient's medical records. The routinely used postoperative patient visit forms were used to collect the presence of postoperative nausea-vomiting, itching, NRS scores, opioid consumption, and further analgesic demands.

Eleven parameters were recorded from the patients' preoperative complete blood count (Sysmex XN-3000). WBC (leukocyte count), RBC (erythrocyte count), PLT (platelet count), NEU (neutrophil count), LYM (lymphocyte count), MONO (monocyte count), EOS (eosinophil count), BASO (basophil count), MPV (mean platelet volume), PCT (percentage of thrombocytes) and PDW (platelet distribution width) were recorded. NLR (neutrophil to lymphocyte ratio) was calculated as absolute neutrophil count/lymphocyte count, PLR (platelet to lymphocyte ratio) as absolute platelet count/lymphocyte count, ELR (eosinophil to lymphocyte ratio) as absolute eosinophil count/lymphocyte count, and SII (systemic immune-inflammation index) as platelet count x neutrophil count/lymphocyte count. The patients' preoperative NLR, PLR, and SII scores at 24 hours were compared to their postoperative analgesic requirements and numerical pain scale (NRS) scores.

The standard protocol for general anesthesia and analgesia was administered as follows. After evaluation and preparation for anesthesia before surgery, patients were transferred to the operating room. Endotracheal intubation was performed after anesthesia was induced using 2 mg/kg propofol, 2 mcg/kg fentanyl, and 0.6 mg/kg rocuronium in the operating room. Anesthesia was maintained with 50%/50% oxygen-air mixture, sevoflurane or desflurane at the appropriate minimum alveolar concentration (MAC), and remifentanil administered intravenously at 0.1-0.5 mcg/kg/min (iv). In accordance with our clinic's standard analgesia procedure, 1 g of intravenous paracetamol, 50 mg of intravenous tramadol, and 20 mg of intravenous tenoxicam were administered during the final 30 minutes of surgery. The patients were extubated after adequate reversal of muscle relaxant. Patients are administered 1 gr iv paracetamol every six hours, 20 mg iv tenoxicam every 12 hours, and tramadol iv patient-controlled analgesia (PCA) device as part of our institute's postoperative analgesic protocol. PCA included 100 ml of normal saline containing 350 mg of tramadol. PCA was set at 20 mg boluses (to be used when NRS ≥4) with a 20-minute lockout time, without basal infusion, and for a maximum dosage of 100 mg per four hours. If, despite, the aforementioned planned analgesia regime and PCA, the patient's NRS was four or above for more than two hours, then 0.05 mg/kg of intravenous morphine was used as rescue analgesia. Patients' 24th postoperative hour NRS scores at rest and during movement, the amount of analgesic administered, the need for additional analgesics, and the occurrence of nausea-vomiting were frequently monitored and documented on the postoperative patient evaluation form.

Statistical analysis

Statistical analysis was performed using SPSS Inc. Released 2009. PASW Statistics for Windows, Version 18.0. Chicago: SPSS Inc. Data are reported as mean, median, standard deviation, and percentage, where appropriate. Comparison of means between groups was compared using the t-test. Chi-square tests were used to compare categorical variables. Correlations were evaluated with the Receiver Operating Characteristics (ROC) curve. AUC (Area Under the Curve) values greater than 0.60 and p < 0.05 were accepted as having statistical significance.

## Results

**Figure 1:**
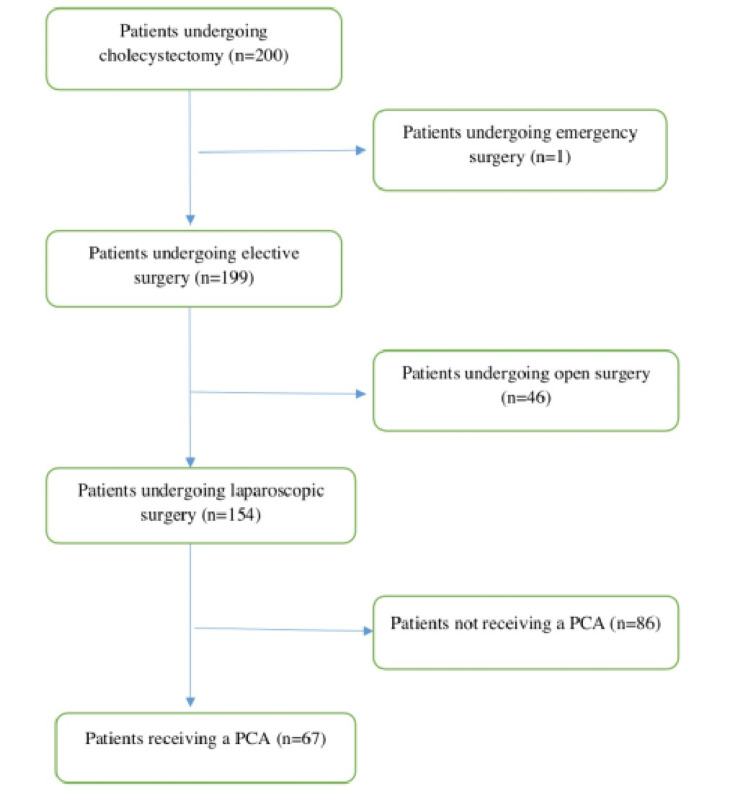
Flowchart of patient inclusion and exclusion to the study n: Number of patients, PCA: Patient controlled analgesia

One hundred and fifty-four patients underwent LC during the study time. Of these, 67 (21 male and 46 female) were included in the study. The flow diagram for patient inclusion is shown in Figure 1.

The average age of patients was 50±13 years. When preoperative hemogram parameters, NRS scores at rest and during movement, and 24-hour tramadol consumption were compared, a statistical relationship was found between pain scores and tramadol use (p=0.0001). There was no relationship between patients' age, surgical time, hemogram parameters, and 24-hour tramadol use (p>0.05) (Table 1).

**Table 1:** Relationship between descriptive data and hemogram parameters and cumulative tramadol use. *p<0.05 “NRS-M: Numeric rating score during movement, NRS-R: Numeric rating score during rest, WBC: Leucocyte count, RBC: Erythrocyte count, PLT: Platelet count, NEU: Neutrophil count, LYM: Lymphocyte count, MONO: Monocyte count, EOS: Eosinophil count, BASO: Basophil count, MPV: Mean platelet volume, PCT: Percentage of thrombocytes, PDW: Platelet distribution width, NLR: Neutrophil to lymphocyte ratio, PLR: Platelet to lymphocyte ratio, ELR: Eosinophil to lymphocyte ratio, SII: Systemic immune-inflammation index”

Variables	vs. cumulative tramadol (n= 67)	
	Pearson coefficient (r)	p
Age	0.012	0.924
Surgical Time (Minutes)	-0.108	0.384
NRS-M	0.455	0.0001*
NRS-R	0.452	0.0001*
WBC	-0.072	0.564
RBC	-0.121	0.33
PLT	-0.151	0.224
NEU	-0.041	0.741
LYM	-0.063	0.614
MONO	0.14	0.258
EOS	-0.039	0.754
BASO	-0.135	0.274
MPV	-0.044	0.726
PCT	-0.187	0.129
PDW	0.134	0.278
NLR	0.106	0.391
PLR	0.032	0.798
SII	-0.013	0.915
ELR	0.021	0.864

Average tramadol consumption over 24 postoperative hours was found to be 190.45±86.85 mg. The patients were separated into two groups based on their 24-hour tramadol consumption: those receiving less than or more than 180 mg (180 mg median value) of tramadol. The two groups were similar with regards to age, gender, ASA classification, length of surgery, NLR, PLR, SII, NRSH, NRSI, WBC, RBC, PLT, NEU, LYM, MONO, EOS, BASO, MPV, PCT, and PDW (p>0.05) (Table 2).

**Table 2:** Demographic and clinical characteristics of patients based on tramadol use. a) Chi-square, Yates (Continuity Correction), b) Chi-square Linear-by-Linear Association, c) Independent Samples Test. NRS-M: Numeric rating score during movement, NRS-R: “Numeric rating score during rest, WBC: Leucocyte count, RBC: Erythrocyte count, PLT: Platelet count, NEU: Neutrophil count, LYM: Lymphocyte count, MONO: Monocyte count, EOS: Eosinophil count, BASO: Basophil count, MPV: Mean platelet volume, PCT: Percentage of thrombocytes, PDW: Platelet distribution width, NLR: Neutrophil to lymphocyte ratio, PLR: Platelet to lymphocyte ratio, SII: Systemic immune-inflammation index”

Variables	Tramadol Use		p		
	0-180 mg (n=35)	< 180mg (n=32)			
				Age. years	50.1±13.4	51.0±14.1	^c^0.79		
Gender. n (%)			^a^0.19		
Male	8 (%22.9)	13 (%40.6)			
Female	27 (%77.1)	19 (%59.4)			
ASA Group. n(%)			^b^0.17		
I	5 (%14.3)	8 (%25.0)			
II	27 (%77.1)	23 (%71.9)			
III	3 (%8.6)	1 (%3.1)			
Operation Time (minutes). average (Range)	47 (30-150)	45 (30-120)	^c^0.27		
NLR	2.0±0.9	2.1±1.2	^c^0.79		
PLR	137.5±53.1	128.6±51.9	^c^0.49		
SII	601.0±329.1	556.4±322.6	^c^0.58		
NRS-M	2.0±1.2	3.1±1.6	^c^0.002		
NRS-R	1.3±0.7	2.2±1.4	^c^0.003		
WBC	7.4±1.7	7.3±2.1	^c^0.82		
RBC	4.8±0.5	4.7±0.4	^c^0.27		
PLT	299.3±99.3	272.0±56.2	^c^0.17		
NEU	4.3±1.3	4.3±1.8	^c^0.95		
LYM	2.3±0.6	2.3±0.8	^c^0.95		
MONO	0.6±0.2	0.7±1.0	^c^0.50		
EOS	0.2±0.1	0.2±0.1	^c^0.24		
BASO	0.1±0.2	0.0±0.0	^c^0.11		
MPV	9.7±1.2	9.7±1.7	^c^0.89		
PCT	0.3±0.1	0.3±0.1	^c^0.12		
PDW	13.2±2.9	14.3±2.6	^c^0.13		

**Figure 2:**
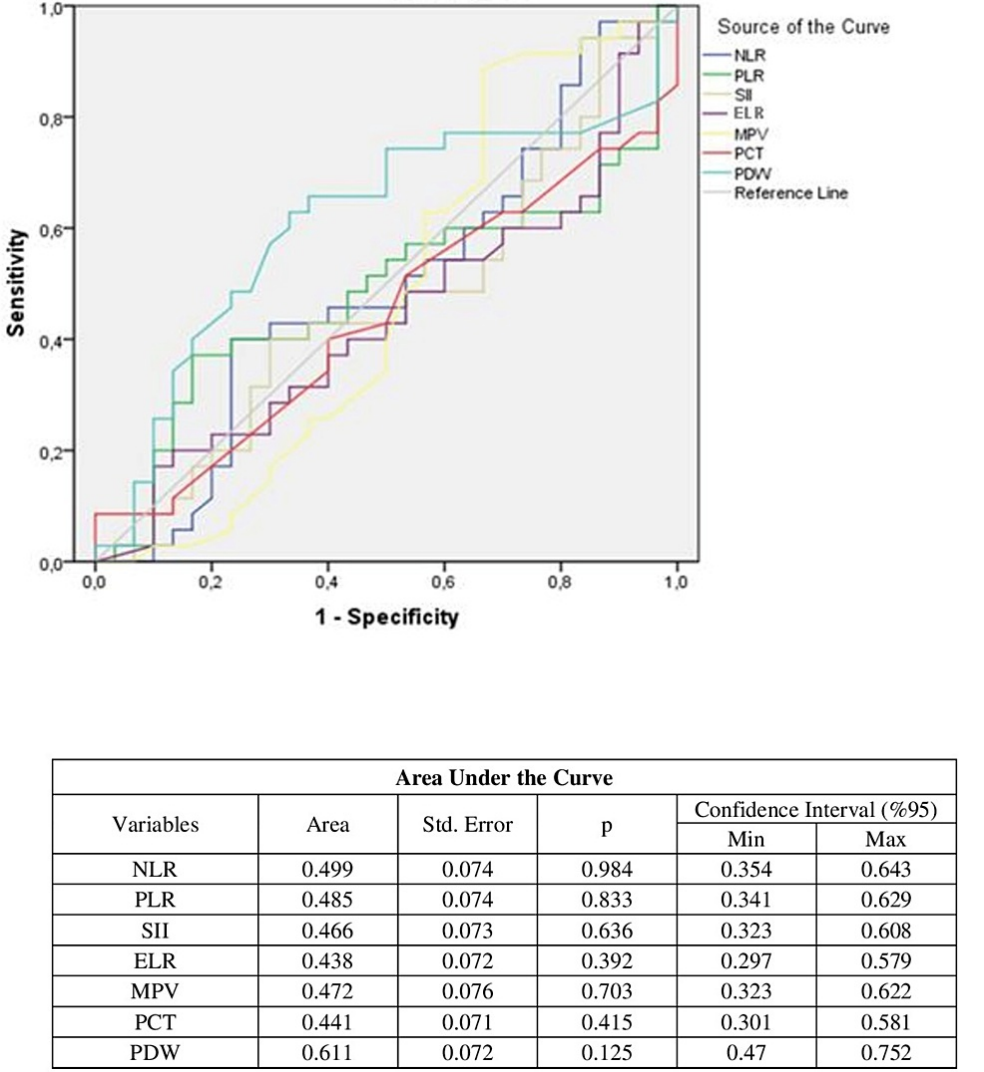
ROC (receiver operating characteristic) curve “NLR: Neutrophil to lymphocyte ratio, PLR: Platelet to lymphocyte ratio, SII: Systemic immune-inflammation index, ELR: Eosinophil to lymphocyte ratio, MPV: Mean platelet volume, PCT: Percentage of thrombocytes, PDW: Platelet distribution width”

No correlation was detected on the ROC curve between NLR, PLR, SII, ELR, MPV, PCT, and postoperative 24-hour tramadol consumption (AUC < 0.6). There was only a statistically significant weak correlation between PDW and postoperative 24-hour tramadol consumption (AUC > 0.6) (Figure 2).

## Discussion

In our study of patients undergoing LC, there was a significant relationship between tramadol use and pain scores on movement and during rest. There was no association between cumulative postoperative tramadol consumption and NRS scores, NLR, PLR, SII values, or other hemogram parameters. A weak correlation was observed between PDW and cumulative 24-hour tramadol consumption (AUC=0.611).

Postoperative pain is an acute status of discomfort that commences with surgical trauma and diminishes as the tissue heals [7]. Acute postoperative pain is a normal response to surgical trauma and increases the risk of wound infection and respiratory/cardiovascular problems, as well as delaying recovery and hospital discharge. Untreated acute pain reduces patient satisfaction, increases morbidity and mortality, and imposes a financial burden on both the patient and the healthcare system [8]. In addition to acute pain, the postoperative period is marked by complex physiological responses involving various systems. In terms of minimizing metabolic and endocrine stress responses, thromboembolic implications, cognitive function protection, mobilization and rehabilitation time, hospital stay and cost, and preventing the development of chronic pain, pain alleviation is significant. Postoperative pain treatment has a substantial impact on the prognosis and quality of life of the patient [7].

The pathophysiological pain classification system identifies nociceptive and neuropathic pathways as mechanisms of pain formation. Nociceptive pain is caused by damage to tissues such as internal organs, muscles, and/or bone, and it is classified into two types: somatic pain and visceral pain. Somatic pain develops as a result of injuries to the skin and musculoskeletal system. Somatic pain is further classified as superficial and deep somatic. For example, pain caused by a minor incision on the skin's surface causes superficial somatic pain, whereas hip fracture leads to deep somatic pain. Visceral pain, also known as referred pain, is discomfort that originates in the internal organs and is difficult to pinpoint [9]. Numerous investigations have demonstrated that pain following LC consists of distinct components, including somatic (incisional) pain, visceral (deep abdominal pain), and referred (shoulder) pain, with varying intensities and durations. In general, the amount and length of post-LC pain vary greatly amongst individuals and are quite unpredictable [2,10-13]. Chemical irritants, rapid stretching of organs, excessive contractions, and diminished blood supply are all causes of visceral pain. Referred pain is felt in a location other than the stimulus site and manifests as shoulder pain in the postoperative phase of LC as a result of carbon dioxide (CO2) gas irritation and exposure to the pressure of the diaphragm muscle and phrenic nerve [2,12-16]. In addition to somatic pain, visceral pain was also prominent in our group of patients undergoing LC.

Several studies, including those regarding postoperative pain, have shown a correlation between various pathologies with hemogram parameters and indexes. In a study involving 140 patients who had orthognathic surgery, Turgut et al. [17] reported a correlation between high NLR scores and postoperative analgesic demand. In a group of 101 patients that underwent total knee arthroplasty and total hip arthroplasty procedures, Canbolat et al. [4] reported that postoperative 48^th^-hour pain scores and NLR were correlated. The outcomes of these investigations are primarily regarding somatic pain. In another study, a substantial association was discovered between propofol injection pain and NLR, PLR, and SII, and these three measures were deemed predictive of propofol injection pain [5].

Another investigation on LC patients also revealed a link between NLR and surgical pain. In their study involving 60 patients who had LC, Daoudia et al. [18] assessed the preoperative pain-related attitudes of the patients and their postoperative analgesic requirements. The authors discovered that patients with high situational pain scale ratings also had high postoperative analgesic needs and that these patients had a low NLR and bad emotional state. However, in this investigation, low NLR values were found to be more predictive than high values.

In a study by Yiğit et al. [19], MPV and PDW values, which are thrombocyte indices, were evaluated in 208 patients with acute appendicitis. The authors demonstrated that platelet indices are not useful in the diagnosis of acute appendicitis due to the numerous factors that influence MPV and PDW. They found that these parameters have no predictive value for the diagnosis of acute appendicitis.

In addition to their uses in predicting postoperative pain, it has been reported that hematological parameters like NLR, PLR, and lymphocyte-monocyte ratio (LMR) obtained from a hemogram are simple tests that can be used as reference indicators in the follow-up of rheumatological diseases to determine the systemic inflammatory [20]. It has also been demonstrated that NLR can be utilized as an indication of mortality in patients undergoing coronary artery bypass graft surgery, especially in the elderly [21].

We believe that our findings can be explained by the fact that both visceral and somatic pain is prominent in patients undergoing LC. In addition to parameters such as NLR, PLR, and SII, that were evaluated in this study and which are especially useful in predicting inflammatory conditions, indices such as fibrinogen-albumin ratio, c-reactive protein (CRP) albumin ratio, albumin-fibrinogen ratio, erythrocyte sedimentation ratio are further biomarkers [22,23] that require studies for their use in predicting for postoperative pain.

Our study's retrospective design is one of its limitations. Propensity score matching could have remedied this issue; however, the number of patients was insufficient. We were only able to include 67 patients as we are unable to offer a PCA device to every patient undergoing LC in our institute. Markers like NLR, PLR, and SII are directly associated with chronic inflammation and can be influenced by a variety of factors such as obesity, dietary habits, smoking, acute infection, etc. To investigate the relationship between logistic regression analysis and independent variables, a prospective study can be done in homogenized groups, or more patients can be included in the study.

## Conclusions

In conclusion, parameters such as NLR, PLR, and SII, which are primarily used to predict the inflammatory response, are also used as simple, inexpensive, and easily accessible markers used to predict clinical course in various clinical scenarios. However, these factors were ineffective in predicting postoperative pain levels and tramadol use following LC. Randomized, controlled clinical trials are required to determine the efficacy of NLR, PLR, SII, and these biomarkers in predicting postoperative pain, particularly in laparoscopic procedures.
